# Optimal Ethanol-Ethiodol Emulsion Ratio in Renal Angiomyolipoma Embolization: A Question that Remains Unanswered

**DOI:** 10.25259/JCIS-21-2019

**Published:** 2019-05-24

**Authors:** Sreeja Sanampudi, Driss Raissi

**Affiliations:** 1 UK Medical Center MN 150, University of Kentucky College of Medicine, University of Kentucky, Lexington, Kentucky, United States.; 2 Department of Radiology, University of Kentucky College of Medicine, Lexington, Kentucky, United States.

**Keywords:** Angiomyolipoma, Emulsion, Ethanol, Ethiodol, Transarterial embolization

## Abstract

The authors, a case series of five cases of angiomyolipomas (AMLs), embolized with a high ethanol:ethiodol ratio of 6:1 emulsion at a single tertiary center. Although ethanol as an embolic agent has been reported in the past for AMLs, much higher ratios of ethiodol were used, and administration is typically performed using an occlusion balloon. Two of the patients were incidentally diagnosed, while the other four were diagnosed after hematuria workup. Of the five patients, only one developed postembolization syndrome. Otherwise, no complications, recurrences, or reinterventions are reported. Our higher ratio seems to allow for adequate radio-opacity of the emulsion with minimal negative dilution effect.

## INTRODUCTION

Angiomyolipomas (AMLs) are benign renal masses consisting of abnormal vascular, adipose, and smooth muscle tissue. Their incidence is <0.3% in the general population.^[1]^ Most AMLs occur sporadically with occasional ones associated with genetic syndromes such as tuberous sclerosis.^[2]^ Sporadic ones tend to occur in an older population and are typically solitary lesions, whereas those associated with tuberous sclerosis tend to be bilateral and multiple. The treatment for AMLs has evolved from nephrectomies to embolization, thereby increasing the amount of spared renal tissue.^[3,4]^ AMLs are typically treated if patients are symptomatic (flank pain, hematuria, anemia, hemorrhagic shock, etc.) or if the AML size is >4 cm to decrease the risk of bleeding and symptoms of mass effect.^[2,4]^ Otherwise, they are managed conservatively.

Transarterial embolization of AMLs is increasingly used thanks to its minimally invasive nature and the significant amount of renal parenchyma spared.^[4]^ Different types of embolic materials are available for AML embolization, including dehydrated ethanol, polyvinyl alcohol, microcoils, N-butyl cyanoacrylate, tris-acryl gelatin microspheres, etc. The most commonly reported ethanol:ethiodol ratios used for AML embolization without using occlusion balloons are 7:3 and 3:1.^[4,5]^ However, a significant number of recurrences were reported in those studies.

Our case series looks at the efficacy and safety of using a higher ratio of 6:1 ethanol:ethiodol emulsion in the embolization of AMLs than previously reported in the literature while maintaining adequate radio-opacity of the emulsion.

## MATERIALS AND METHODS

**Figure 1:**
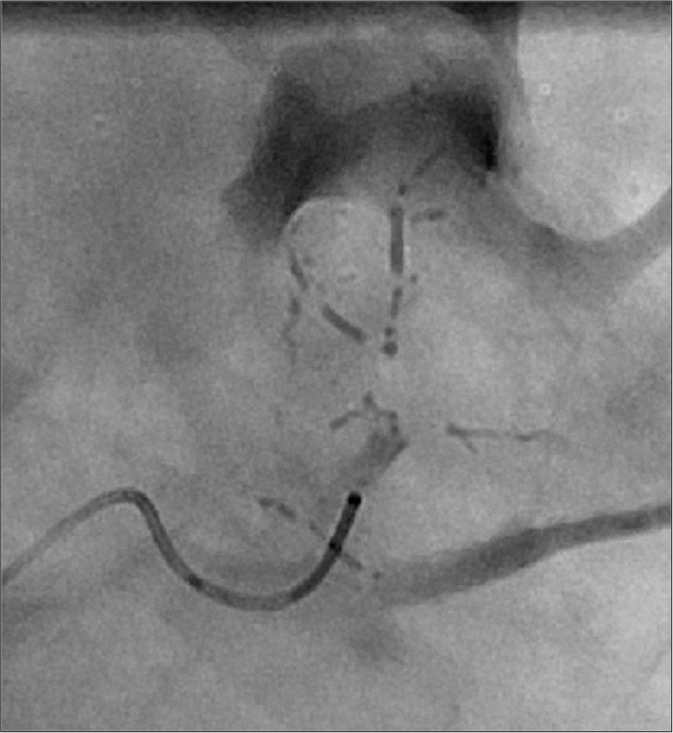
Fluoroscopic spot image of subsegmental renal artery being embolized with ethanol:ethiodol in the form of “oil bullets.” The 6:1 ratio of ethanol:ethiodol offers adequate radio-opacity while providing effective embolic properties.

**Figure 2:**
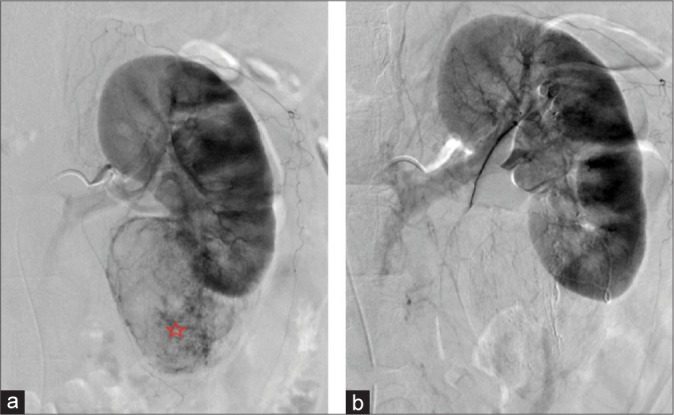
A 64-year-old woman with an incidental angiomyolipoma (AML) found during imaging for bowel resection. (a) Digital subtraction angiography of the left renal artery demonstrating the contrast-enhancing AML (red star) off the inferior pole of the left kidney. (b) Digital subtraction angiography of left renal artery postembolization demonstrating lack of enhancement of AML indicating technical success of embolization procedure.

Institutional Review Board approval of this case series was obtained. The AMLs were diagnosed with the use of contrast-enhanced computed tomography (CT) and/or magnetic resonance imaging of the abdomen. The embolization procedures were performed in the angiography suite with the patients either under general anesthesia or conscious sedation. The common femoral artery was accessed using the Seldinger technique. The renal artery is selected, and digital subtraction angiography of the vessel is performed. A 2.8-French microcatheter is used to select the distal segmental branches supplying the AML. The patients were given 200 mg intra-arterial nitroglycerin and 5 mg nifedipine intravenously to prevent vasospasm. Ethiodol (Guerbet^®^, Sulzbach, Germany) was mixed with dehydrated ethanol through standard Tessari method using 6:1 ratio to produce a homogeneous emulsion. Embolization of the arterial branches supplying the AML using ethanol:ethiodol emulsion was performed until stasis was achieved. Figure 1 postembolization angiography of the main renal artery was performed. Figure 2a and b demonstrates the pre- and postembolization angiography images of patient 1. Complete stasis of flow in the target vessels was considered technical success, while tumor shrinkage, lack of complications, AML bleeding, or reinterventions was considered clinical success.

This case series includes five patients, all of whom had AMLs occur sporadically and not in association with a genetic syndrome.

### Study design

A case series retrospectively reviewing five fluoroscopy-guided AML embolization cases performed from January 01, 2016, to August 30, 2018, using a fixed ratio of ethanol:ethiodol 6:1 emulsion as the only embolic agent. Five cases met our inclusion criteria. AML embolizations performed with other embolic agents were excluded. A single operator with over 5 years’ experience performed all cases.

### Cases report 1–2

**Figure 3:**
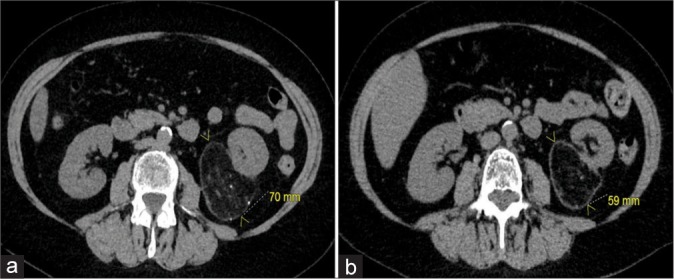
A 64-year-old woman with an incidental angiomyolipoma (AML). (a) Axial computed tomography (CT) of the abdomen and pelvis demonstrating the AML off the inferior pole of the left kidney measuring 70 mm. (b) Axial CT of the abdomen and pelvis at an 18-month follow-up showing shrinkage of the AML measuring 59 mm.

**Figure 4:**
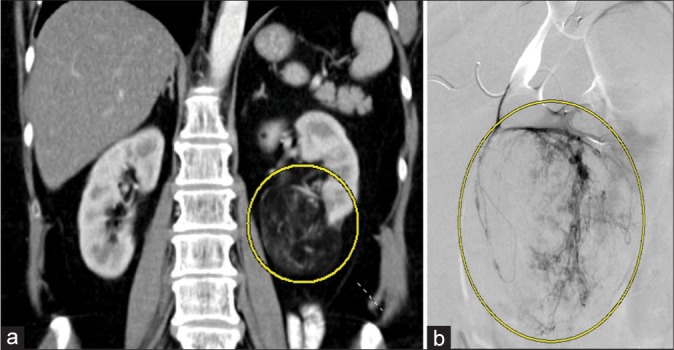
A 64-year-old woman with an incidental angiomyolipoma (AML). (a) Coronal computed tomography of the abdomen and pelvis demonstrating the AML (yellow circle) off the inferior pole of left kidney measuring 70 mm. (b) Digital subtraction angiography of a subsegmental renal artery demonstrating contrast enhancement of the AML off the inferior pole of the left kidney.

Two (ages 64 and 71) of the five patients with AMLs were diagnosed incidentally while undergoing evaluation for gastrointestinal bleeding and bowel resection, respectively. Both of these patients were female, with the AML located in the inferior pole of the left kidney, and the size ranging from 6.9 to 7 cm. Patient 1 had no complications, and the AML decreased in size by 16% at the end of 18 months as demonstrated in Figure 3. Additional images of the AML are demonstrated with coronal CT and digital subtraction angiography [Figure 4a and b]. Both patients were embolized with 6:1 ethanol:ethiodol emulsion. The second patient was lost to follow-up (f/u).

### Cases report 3–5

**Figure 5:**
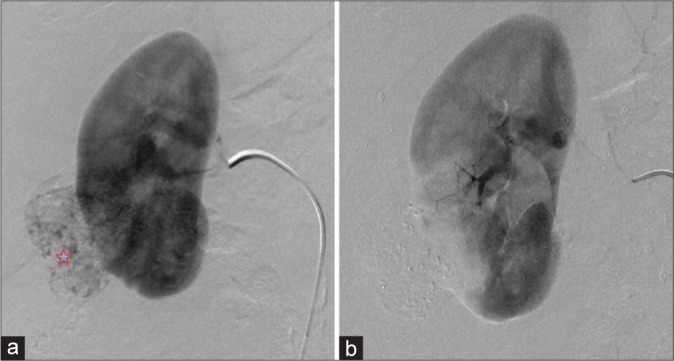
A 57-year-old female presenting with hematuria with subsequent diagnosis of angiomyolipoma (AML). (a) Digital subtraction angiography of the right renal artery demonstrating the contrast-enhancing AML (red star) off the inferior pole of right kidney. (b) Digital subtraction angiography of the right renal artery postembolization demonstrating lack of enhancement of AML indicating technical success of AML embolization.

Patients 3–5 with AMLs were diagnosed after hematuria workup. All three patients were female. Two of the masses are located in the inferior pole of the right kidney and the third one in superior pole of the left kidney. The masses ranged in size from 4.8 to 6 cm. All three patients were embolized with ethanol:ethiodol 6:1 emulsion. With the exception of patient 3, the patients had a decrease in the size of AML postembolization ranging from 20% to 40% with a greater reduction seen at longer f/u durations. None of the patients developed complications. The AML shown by digital subtraction angiography is represented in Figure 5a and b demonstrating pre- and postembolization.

The patient characteristics, complications, f/u, and procedure details are listed in Table 1.

**Table 1:** Characteristics of angiomyoplipoma, procedure information, and complications.

Patient	Age	Diagnosis	Location	Preprocedure size (cm)	Size (cm) at last follow-up	Follow-up duration (months)	Fluoroscopy time (min)	Quantity of embolic material	Complications	Reintervention
1	64	Incidental	Left inferior pole	7	5.9	18	10.5	2.2 ml of ethanol: ethiodol emulsion	Postembolization syndrome - treated with acetaminophen and NSAIDs	No
2	71	Incidental	Left inferior pole	6.9	N/A	Loss to follow-up	13.6	2 ml of ethanol: ethiodol emulsion	None	No
3	63	Hematuria	Left superior pole	5	5	6	6.6	3 ml of ethanol: ethiodol emulsion	None	No
4	66	Hematuria	Right inferior pole	4.8	2.9	27	5.7	1.8 ml of ethanol: ethiodol emulsion	None	No
5	57	Hematuria	Right inferior pole	6	4.8	6	8	3 ml of ethanol: ethiodol emulsion	None	No

N/A: Not available, NSAIDs: Nonsteroidal anti-inflammatory drugs

## DISCUSSION

AMLs are benign masses that consist of fatty, vascular, and muscular tissues. Several therapies are available for AMLs ranging from partial nephrectomies and nephron-sparing surgeries to medical management with mTOR inhibitors and antiangiogenic medications. Although partial nephrectomies are associated with lower rates of recurrence, they have higher rates of complications.^[6]^ Of the surgical options, minimally invasive nephron-sparing surgery remains a viable option for smaller AMLs with low rates of recurrence and complications.^[5]^ Medical management is typically reserved for patients who are not candidates for nephron-sparing surgery and embolization.^[6]^ Transarterial embolization has become the first-line treatments due to its minimally invasive nature, fewer hemorrhagic complications, tumor shrinkage, kidney function preservation, and lower mortality rates.^[7]^ A self-limiting postembolization syndrome (fever, leukocytosis, pain, nausea, and vomiting) is relatively common and occurred in ~36% of patients in one review article.^[7]^

The first transarterial embolization of AML dates back to 1977 with the use of gelfoam.^[8]^ Since then, a variety of embolic agents have been used including ethanol, polyvinyl alcohol particles, gelfoam, N-butyl cyanoacrylate, etc. The use of combination of embolic materials was more common than single agent alone according to Murray *et* *al*.^[7]^ This practice was followed by the use of ethanol as monotherapy. Of the 31 studies that Murray *et* *al*. looked at, only seven of them used ethanol alone resulting in a technical success rate of 95.5% and tumor reduction of 2.3 cm compared to six studies that used two or more embolic materials and had a technical success rate of 95% and mean size reduction of 4.6 cm.^[7]^

However, when ethanol was used, most employed an occlusive balloon to avoid reflux and nontarget embolization.^[9]^ The use of occlusive balloons increases the risk of aneurysmal rupture due to increasing pressure during administration and makes it more difficult to pursue superselective catheterization.^[9]^ The first reported use of ethanol:ethiodol emulsion without an occlusive balloon was reported by Park *et* *al*.^[10]^

The ratios of ethanol:ethiodol emulsion reported in the literature are predominantly 3:1 or 7:3 with the use of occlusion balloons being the most common practice.^[4,5,9]^ In the past, symptom and tumor recurrence was often associated with AMLs occurring in conjunction with tuberous sclerosis.^[4,5]^ Kothary *et* *al*. used a 7:3 ratio of ethanol:ethiodol and reported a median f/u of 51.5 months with 100% success in sporadic AMLs, but a 57.1% in AMLs that occurred in association with tuberous sclerosis.^[5]^ Lee *et* *al*. utilized a 3:1 ratio of ethanol:ethiodol without the use of occlusion balloon and reported recurrence only in 2 of their 15 patients.^[9]^ Similar results were reported by Bishay *et* *al*. who used a 7:3 ratio of ethanol:ethiodol; however, they used occlusion balloons with a recurrence of 2 of 16 patients.^[4]^ We opine that a 10%–12% recurrence rate in these two studies is rather high and could be caused by the overdilution effect from the relatively low ratios of their ethanol:ethiodol emulsion. Hence, we selected a 6:1 ethanol:ethiodol ratio in an attempt to increase the emulsion’s ethanol embolic effect while maintaining an acceptable radio-opacity.

**Figure 6:**
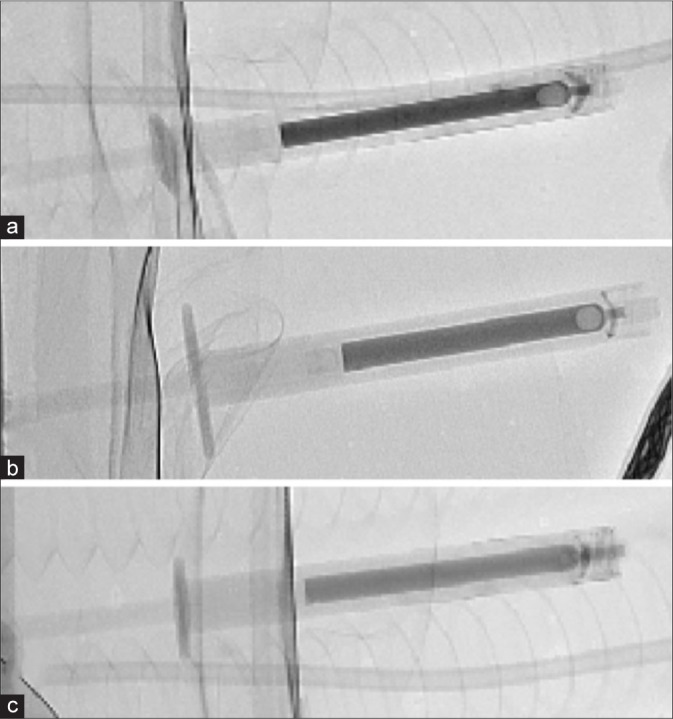
Fluoroscopy image of various ethanol:ethiodol ratios. (a) The ethanol:ethiodol ratio is 4:1 demonstrating highest level of radio-opacity. (b) The ethanol:ethiodol ratio is at 6:1. (c) The ethanol:ethiodol ratio is 9:1 with the least level of radio-opacity.

Dehydrated ethanol has several advantages over other embolic agents in AML embolization, being a low viscosity liquid, it diffuses very distally and deep into the capillary bed of tumor causing irreversible endothelial damage and tumor necrosis.^[10]^ The addition of ethiodol to the mixture allows for visualization of the embolic material, allowing the operator to recognize reflux and avoid nontarget embolization.^[1]^ However, in the pursuit of acceptable radio-opacity, overdilution of the ethanol with ethiodol can lower its efficacy and may increase reintervention rates. On the other hand, underdilution can result in poor visualization of ethanol and inadvertent nontarget embolization. In our experience, the ratio of 6:1 ethanol:ethiodol can offer the optimal balance of proper radio-opacity and maximal embolic effect in the treatment of AMLs. The volumes of ethanol:ethiodol that we have used are demonstrated in Figure 6.

Our case series is indicative of short-term technical and clinical success using a higher ethanol:ethiodol ratio for AML embolization without the need for balloon occlusion than previously reported in the literature. None of the five cases had any regrowth or AML bleeding events with f/u ranging from 6 to 27 months with the exception of one patient who was lost to f/u.

The only reported complication is postembolization syndrome in a single patient presenting 2 days after the procedure. The patient’s symptoms were managed conservatively with NSAIDs and acetaminophen with complete resolution in 3 days. Our conclusions, while offering promising preliminary data are limited by the inherent nature of any case series with its retrospective data and small sample size. This proposed ethanol:ethiodol ratio needs further validation with a prospective study and/or a larger sample size with special emphasis on using a higher ethanol ratio for AML embolization.

## CONCLUSION

Ethanol:ethiodol at a ratio of 6:1 emulsion appears to be safe and effective for AML embolization, allowing for adequate radio-opacity under fluoroscopy while using a relatively high ratio of ethanol to ensure optimal embolization of the AML and with no recurrences found to date in our series. However, a large retrospective or preferably prospective study with long-term f/u is needed to validate our findings and answer our opening question regarding the optimal ethanol:ethiodol ratio for AML embolization.
